# Searching for the one

**DOI:** 10.7554/eLife.32016

**Published:** 2017-09-28

**Authors:** Helga Groll

**Affiliations:** eLifeCambridgeUnited Kingdom

**Keywords:** Peer review, careers in science, gender bias, workforce diversity

## Abstract

The views of peers are important when applying for a faculty position, but so are research plans and being a good 'fit'. Many universities are also trying to reduce bias in their recruitment processes.

The chances of someone with a PhD in the life sciences landing a permanent job in a university are low. According to a report published by the Royal Society in the UK in 2010, only 3.5% of science PhDs end up with such a job, although 17% have research jobs outside a university. This inevitably means that any university looking to recruit new faculty in the life sciences is likely to be overwhelmed with applications, especially in North America and Europe. How do they go about selecting one candidate from the hundreds who apply?

When the Department of Biology at the University of Washington in Seattle advertises to fill a faculty position, it typically receives between 500 and 1,000 applications. Each of these applications will usually include a CV, a publication list, a 200-word statement about the applicant's research plans, a 200-word statement about how their research will fit with other work in the department, and letters of recommendation from leading researchers in the field. The first step in selecting which applicants are invited to Seattle for an interview involves every faculty member screening the statements about research plans and fit to the department. "This typically reduces the list by about half," says Toby Bradshaw, chair of the department.

Screening the CVs and publication lists then reduces the list to about 100, and going through the letters of recommendation reduces it to about 40. So what does the department look for in a publication list? "Big questions, interesting approaches, collaborative and integrative work published in good journals, but a candidate doesn't have to have a Nature or Science paper," says Bradshaw. "However, letters of recommendation have a huge influence."

The remaining candidates are then interviewed by Skype, and between 10 and 20 will be invited to the campus for interview. To be offered the job a candidate will need to convince the search committee that they are "asking biologically interesting questions and taking innovative approaches", and that they will be a good fit to the department. It is also important, adds Bradshaw, to "appreciate the breadth of departmental research and be excited about teaching."

The University of Ottawa in Canada also receives hundreds of applications when it advertises for a faculty position. "Each department has its own method for short-listing candidates," says Steve Perry, Dean of the Faculty of Science at the university, "but, typically, our search committee members are asked to independently rank the top 15 candidates." These rankings are then combined to create a draft short list that is discussed and possibly adjusted by the search committee. The committee then requests letters of recommendation for the top 15 or so applicants. "With these rankings and letters in hand, the committee members then engage in discussion to agree on a list of the top 5–6 candidates."

Perry says that "numerous factors" are considered when ranking candidates, including bibliometric data, reference letters, area of research and fit with the department, publications list (including quality and number of publications, quality of journals, and role in multi-authored publications), research proposal and teaching statements. Awards, honors, years to complete doctorate and years of postdoctoral experience are also considered.

Letters of recommendation have a huge influence

What attributes are the search committee looking for during the interview process? "Excellence in research and teaching, although the latter is not easy to evaluate, effective communication skills, and a well thought-out research plan for the first five years are important," says Perry. Collegiality and an ability to form collaborations within the department are also important, as are ambition and the potential to bring in external funding. "Our recruitment process focuses extensively on research," he adds. "I have been pushing for a more formalized way to judge teaching – such as mock lectures – but to date there is not a standardized policy within the Faculty".

The need to be a good fit and a good colleague is also important at smaller universities. "We look for versatility and attitude," according to an assistant professor at a public university in the midwest who prefers to remain anonymous. "We are a small school and cannot accommodate large and expensive research programs. The candidate must have a clear idea of how they will capitalize on the opportunities (and overcome the challenges) that our department will present to them. The successful candidate will also need to be a decent human being and care about their students, their colleagues and the department – not just about themselves."

In Europe, the Ludwig-Maximilians-Universität (LMU) in Munich is experimenting with a new approach to the recruitment of faculty by encouraging junior researchers who have been awarded Starting Grants by the European Research Council (ERC) to apply for tenure track professorships. According to the LMU website, the appointment "requires the successful completion of a selection procedure (analogous to that for standard faculty appointments) by the respective Faculty at LMU and a successful application for an ERC Starting Grant with LMU as the host institution."

In a blogpost published at the end of 2015, Sean Eddy of Harvard University gave an indication of the amount of time that existing faculty members spend when recruiting new faculty. "The first step is affectionately, brutally dubbed 'triage'," wrote Eddy, who was co-chair of the search committee for a position in systems biology that attracted 182 applications. "Every application gets read, albeit quickly, by three of us. I spend about 10 minutes on each application. You can do the math: it works out to about 30 hours, total."

The competition between candidates is less intense in China, with universities competing with each other to attract the best candidates. "In Chinese universities, we have enough positions for talented young researchers with a good academic background," says Xinliang Zhang, Dean of the School of Optical and Electronic Information at the Huazhong University of Science and Technology (HUST). "In our school, candidates need to have at least two papers in peer-reviewed journals that are widely recognized by the international community." According to Zhang, candidates with papers in well known journals, such as Science or Nature, are "very welcome" at most Chinese universities. "In my opinion, this is not a good thing," says Zhang, "because we cannot evaluate different candidates from different fields with the same criterion. Researchers in some disciplines, such as electronic engineering and computer science, find it more difficult to publish papers in well known journals than researchers in other areas, such as biology or medicine."

While scientists go to great lengths to avoid potential sources of bias in their experiments, the relatively low numbers of women holding senior positions in universities suggests that, in the past at least, selection committees may have been prone to gender bias. However, many universities are taking action to eliminate, or at least reduce, gender bias.

The University of Washington, for example, provides staff with implicit bias training: moreover, the first name is redacted from the CVs to reduce gender bias, and all applications are reviewed by the Diversity and Equity Committee. And when a faculty position is offered to a man at the University of Ottawa, the relevant dean and departmental chair must justify to the Vice-President Academic and Provost of the university why a woman was not selected. "Gender bias is a huge issue that we are currently struggling with at Ottawa," says Steve Perry. "Ultimately, all members of the department vote on the proposed top candidate. We have considered trying to redact the applications to make them gender neutral to eliminate unconscious bias but there does not seem to be an easy solution."

Juan Carlos Reboreda, Dean of the Faculty of Exact and Natural Sciences at the University of Buenos Aires in Argentina, agrees: "It has been shown that there is a bias towards men in science, and that men tend to favor men over women." In an effort to combat gender bias, Reboreda's faculty tries to have equal numbers of men and women on interview panels. Recruiting researchers from outside Argentina can also be a problem for Reboreda, partly because salaries are higher in the other South American countries. "Salaries are not attractive enough to get people from Europe or the US," he says. "We mainly export scientists, rather than import them."

At Harvard, Eddy was worried that people who were highly qualified for the position did not apply: "Our applicant pool starts out biased: it’s only 21% female, and it’s only 5% underrepresented minority." To address this and to counter bias in the selection process, the female and underrepresented minority applications were read and ranked separately from the other applications, and the search committee then sought to interleave the two sets of rankings "as objectively as possible".

The search for a job can, as Eddy put it, be brutal. Most researchers know what it is like to have papers accepted for publication in a journal, but only a small fraction will ever know what it is like to land a faculty position in a university. So good luck to anyone going down this path – you will need it!

## Note

This Feature Article is part of a collection of articles on peer review.

